# Acute Hypersensitivity of Pluripotent Testicular Cancer-Derived Embryonal Carcinoma to Low-Dose 5-Aza Deoxycytidine Is Associated with Global DNA Damage-Associated p53 Activation, Anti-Pluripotency and DNA Demethylation

**DOI:** 10.1371/journal.pone.0053003

**Published:** 2012-12-27

**Authors:** Bijesh K. Biswal, Maroun J. Beyrouthy, Mary P. Hever-Jardine, David Armstrong, Craig R. Tomlinson, Brock C. Christensen, Carmen J. Marsit, Michael J. Spinella

**Affiliations:** 1 Department of Pharmacology and Toxicology, Geisel School of Medicine at Dartmouth, Hanover, New Hampshire, United States of America; 2 Community and Family Medicine, Geisel School of Medicine at Dartmouth, Hanover, New Hampshire, United States of America; 3 Norris Cotton Cancer Center, Geisel School of Medicine at Dartmouth, Hanover, New Hampshire, United States of America; Kinghorn Cancer Centre, Garvan Institute of Medical Research, Australia

## Abstract

Human embryonal carcinoma (EC) cells are the stem cells of nonseminoma testicular germ cells tumors (TGCTs) and share remarkable similarities to human embryonic stem (ES) cells. In prior work we found that EC cells are hypersensitive to low nanomolar doses of 5-aza deoxycytidine (5-aza) and that this hypersensitivity partially depended on unusually high levels of the DNA methyltransferase, DNMT3B. We show here that low-dose 5-aza treatment results in DNA damage and induction of p53 in NT2/D1 cells. In addition, low-dose 5-aza results in global and gene specific promoter DNA hypomethylation. Low-dose 5-aza induces a p53 transcriptional signature distinct from that induced with cisplatin in NT2/D1 cells and also uniquely downregulates genes associated with pluripotency including NANOG, SOX2, GDF3 and Myc target genes. Changes in the p53 and pluripotency signatures with 5-aza were to a large extent dependent on high levels of DNMT3B. In contrast to the majority of p53 target genes upregulated by 5-aza that did not show DNA hypomethylation, several other genes induced with 5-aza had corresponding decreases in promoter methylation. These genes include RIN1, SOX15, GPER, and TLR4 and are novel candidate tumors suppressors in TGCTs. Our studies suggest that the hypersensitivity of NT2/D1 cells to low-dose 5-aza is multifactorial and involves the combined activation of p53 targets, repression of pluripotency genes, and activation of genes repressed by DNA methylation. Low-dose 5-aza therapy may be a general strategy to treat those tumors that are sustained by cells with embryonic stem-like properties.

GEO number for the microarray data: GSE42647.

## Introduction

Testicular germ cell tumors (TGCTs) are responsive to cisplatin-based therapy, even when metastatic [1]. However, 15–20% of patients are refractory to treatment or undergo late relapse and cisplatin-based therapies are associated with life-long toxicities [2], [3]. These represent important clinical issues to overcome with improved therapies. Pluripotent embryonal carcinoma (EC) cells are proposed to represent TGCT stem cells and to be the malignant counterparts to embryonic stem (ES) cells [4], [5]. There is recent evidence to suggest that ES/EC cells are similar to undifferentiated somatic cancers and cancer stem cells but dissimilar to normal adult tissue stem cells [6]–[9]. Thus, strategies devised to target EC cells may have therapeutic utility toward somatic cancer stem cells that possess “ES-like” signatures while sparing normal adult stem cells.

Human cancers have global DNA hypomethylation including hypomethylation of repetitive elements coupled with hypermethylation of CpG islands at specific tumor suppressor gene promoters [10]. There are three main DNA methyltransferases (DNMTs) in mammals, DNMT1, DNMT3A and DNMT3B. DNMT1 is mainly responsible for maintenance DNA methylation while DNMT3A and DNMT3B mediate *de novo* methylation during development [10]. Pluripotent cells including EC express high levels of DNMT3B and recent genome-wide DNA and histone methylation analyses suggest that pluripotent cells are in unique epigenetic states compared to differentiated somatic cells [11]–[16].

Potent inhibitors of DNA methylation are the nucleoside analogs 5-aza-deoxycytidine (5-aza) and 5-aza-cytidine [10]. Notably, 5-aza becomes incorporated into DNA and mediates covalent adduct formation with DNMTs. This is proposed to result in effective inhibition of DNA methylation [17]. The mechanism by which 5-aza elicits anticancer effects is controversial. One mechanism, especially proposed for lower doses of 5-aza, involves demethylation and re-expression of tumor suppressor genes [18]. Other mechanisms involve apoptosis following direct or indirect 5-aza-meditated DNA-damage [19], [20]. We previously discovered that several distinct EC cell lines, even those resistant to cisplatin, are acutely sensitive to very low (<10 nM) doses of 5-aza compared to various somatic tumor cell lines [21]. This was associated with extremely high levels of DNMT3B in EC cells compared to the levels in somatic tumor cells. Notably, DNMT3B knockdown resulted in substantial resistance to 5-aza in NT2/D1 EC cells, suggesting that 5-aza hypersensitivity in EC is mechanistically linked to high levels of DNMT3B [21].

Clinical data is emerging that 5-aza at lower doses produces delayed, long-term antitumor responses *in vivo* and *in vitro* in somatic tumor cells [22]–[24]. The kinetics of these low-dose 5-aza responses is consistent with targeting of tumor initiating or cancer stem cells. In the present study we discover that in malignant stem-like NT2/D1 cells low-dose 5-aza elicits a distinct DNMT3B-dependent genome-wide activation of p53 target genes together with both DNA damage and global DNA demethylation of specific gene promoters. Further, 5-aza mediates an early and dramatic DNMT3B-dependent downregulation of pluripotency genes in NT2/D1 cells.

## Results

### Low-dose 5-aza induces an acute apoptotic response in cisplatin-sensitive and -resistant testicular cancer cells and can decrease global DNA methylation

**Figure 1:**
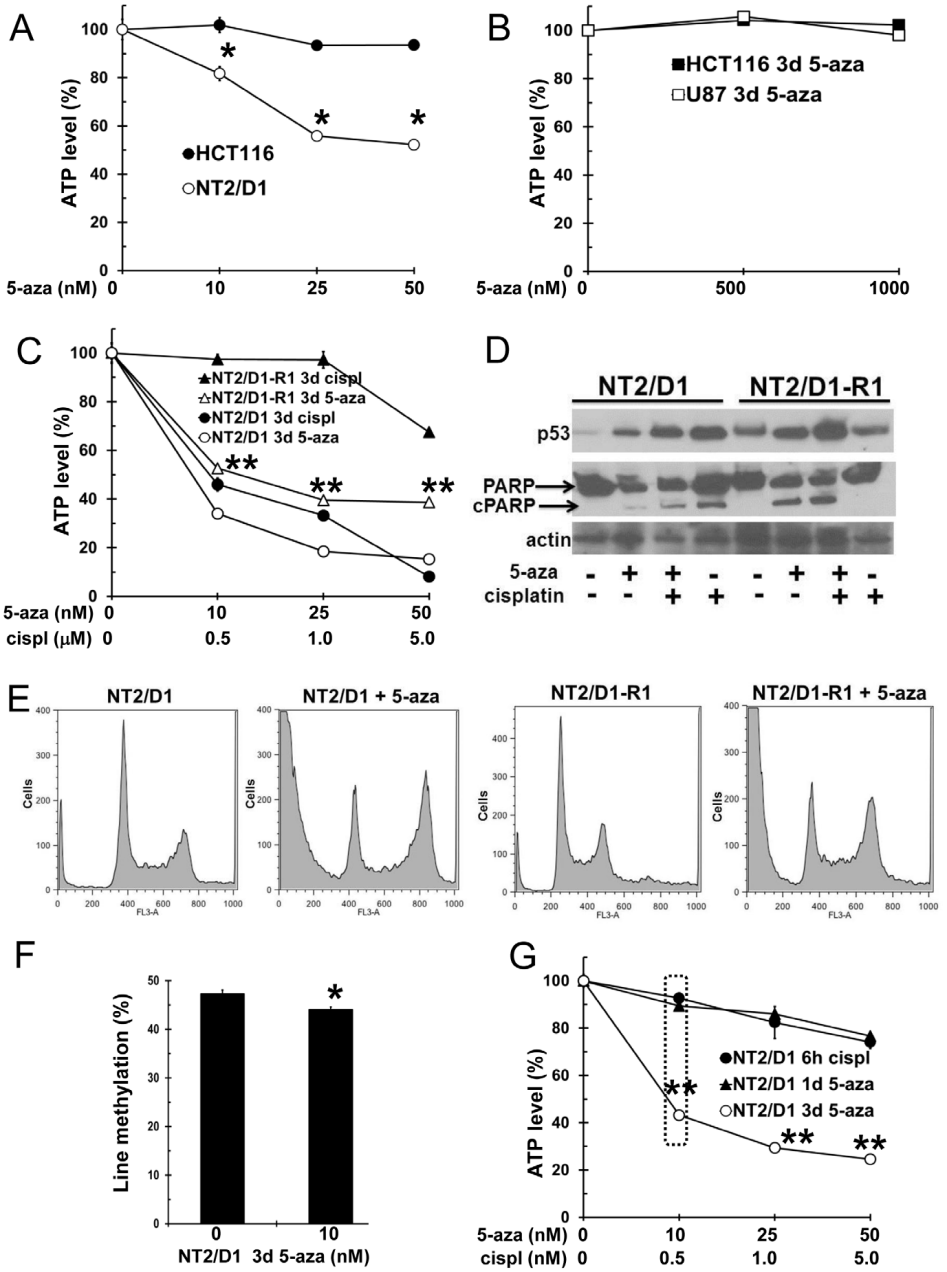
Low-dose 5-aza induces an acute death response in cisplatin sensitive and resistant EC cells. **A–B**, The EC line NT2/D1 but not colon cancer HCT116 cells or glioblastoma U87 cells are sensitive to low-dose 5-aza. 5-aza was added for 3 days to exponentially growing cultures. Viable cell growth and survival were measured. **C**, Cisplatin resistant NT2/D1-R1 cells remain sensitive to low-dose 5-aza. NT2/D1 and NT2/D1-R1 cells were treated with cisplatin for 6 hours and assayed 3 days later or 5-aza was added for 3 days. **D**, 5-aza induces p53 protein and induces cell death in NT2/D1 and NT2/D1-R1 cells while cisplatin induces p53 protein and cell death only in NT2/D1 cells. Cell were treated with 10 nM 5-aza for 3 days or 1.0 µM cisplatin for 12 hours or the combination and Western analysis performed for p53 and PARP. The arrow indicates cleaved 85 KD PARP that is indicative of apoptosis. **E**, Treatment with 5-aza results in cell death and G2 cell cycle arrest in NT2/D1 and NT2/D1-R1 cells. Cells were treated with 10 nM 5-aza for 3 days. Substantial cells in subG1 are indicative of cell death. **F**, Low-dose 5-aza induced global demethylation in NT2/D1 and colon cancer HCT116 cells. Cells were treated with the indicated does of 5-aza for 3 days and methylation of LINE-1 elements were measured by DNA bisulfite-pyrosequencing. **G**, Viable cell growth and survival of treatment protocols for subsequent expression array analysis. NT2/D1 cells were treated with indicated doses of 5-aza added for 1 or 3 days before harvest or indicated doses of cisplatin for 6 hours follows by 24 hour harvest. Dotted box indicates cell treatments used for expression arrays. All bars and data points are the average of biological triplicates. Error bars are standard deviation and are within the size of the symbols in most cases. * = p<0.05; ** = p<0.005 compared to untreated controls.

In prior work we established that several testicular cancer-derived embryonal carcinoma (EC) cell lines including NT2/D1 cells are acutely sensitive to low doses of 5-aza, a property not shared by various somatic tumor cell lines [21]. There is a decrease in the number of NT2/D1 cells after a 3 day treatment with 5-aza at doses as low as 10 nM while HCT116 colon cancer and U87 glioblastoma cells are unaffected at doses as high as 1 µM (Fig. 1A and Fig. 1B). Cisplatin resistant EC cells including the cisplatin resistant line, NT2/D1-R1, retain exquisite sensitivity to low-dose 5-aza [21] (Fig. 1C). Three day low-dose 5-aza treatment results in p53 induction and responses consistent with apoptosis in NT2/D1 and NT2/D1-R1 cells as determined by Western analysis, poly(ADP-ribose) polymerase (PARP) cleavage and induction of cells with sub G1 DNA content while cisplatin induces these responses only in cisplatin-sensitive NT2/D1 cells (Fig. 1D and Fig. 1E). The 5-aza mediated PARP cleavage fragment is predominantly an 85 KD band consistent with apoptosis. Further, induction of p53 protein by 5-aza is not associated with increased p53 mRNA suggesting that 5-aza induces p53 stability (data now shown). Additionally, 3 day 5-aza treatment resulted in a higher percentage of cells in the G2 phase of the cell cycle compared to untreated cells as can be seen by a more prominent G2 peak and a less prominent G1 peak (Fig. 1E). Notably, NT2/D1-R1 cells are co-resistant to a variety of conventional DNA damaging chemotherapeutics including etoposide, vinblastine and doxorubicin (data not shown); suggesting that the 5-aza response in NT2/D1 and NT2/D1-R1 cells is mechanistically distinct from the classical DNA damage response. Low dose 5-aza also resulted in a significant reduction in global DNA methylation in NT2/D1 cells as assessed by repetitive long interspersed nuclear element-1 (LINE-1) bisulfite pyrosequencing (Fig. 1F).

### Low-dose 5-aza induces an early, robust, and unique reprogramming of gene expression in NT2/D1 cells

We conducted a series of microarray-based gene expression analyses that compared gene expression changes in NT2/D1 cells treated with 10 nM 5-aza for 1 or 3 days to NT2/D1 cells treated with 0.5 µM cisplatin for 6 hours followed by a 24 hour recovery. The cisplatin protocol was identical to our previously reported study [25]. While effects on cell viability and proliferation are minimal after 1 day of cisplatin treatment (Fig. 1G), robust anti-proliferation and cell death are seen 2 days later (Fig. 1C and Fig. 1D). Three day 5-aza was chosen since demethylation is expected to require several cell doublings for incorporation of the 5-aza analog into DNA and NT2/D1 cells double every 24 hours. A 1 day 10 nM 5-aza treatment was included to assess early effects of 5-aza. In the majority of cases approximately 60% cell death was seen after 3 days of 10 nM 5-aza treatment of NT2/D1 cells (Fig. 1C and Fig. 1G), yet for unclear reasons, an occasional decrease in the relative extend of cell death was noted (Fig. 1A).

**Figure 2:**
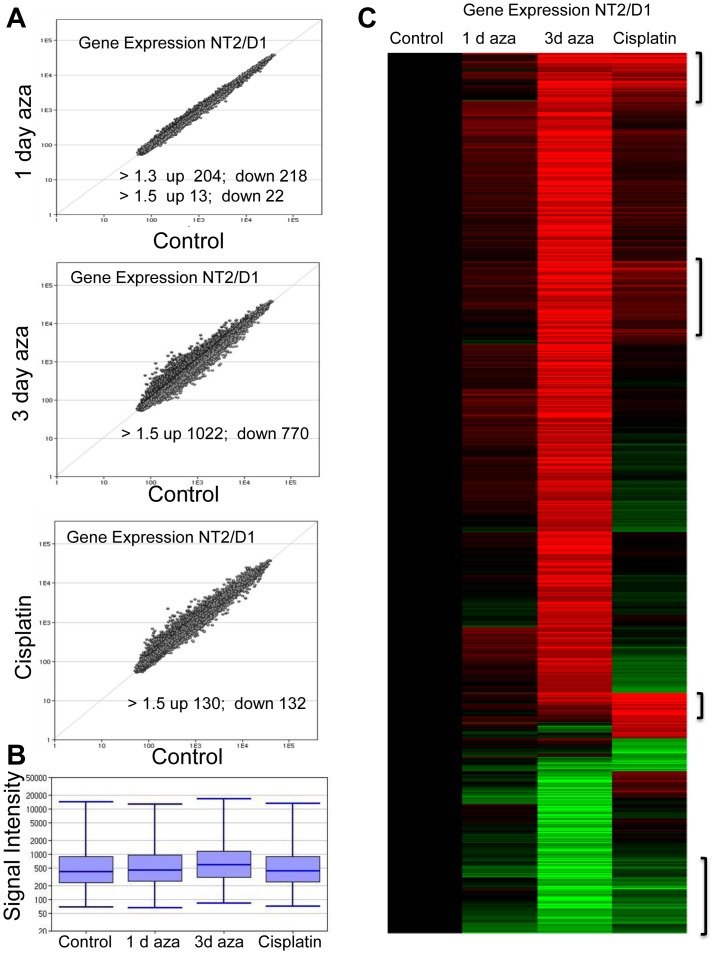
Low-dose 5-aza induces an early, robust, and unique reprogramming of gene expression in NT2/D1 cells. **A**, Scatter plot of microarray gene expression differences in NT2/D1 cells. Each point represents the average of 3 biological replicates. Points above the diagonal line represent genes upregulated and points below the diagonal line repressed with 1 day 5-aza treatment (top), 3 day 5-aza treatment (middle), and cisplatin treatment (bottom), compared to control. The number of altered genes above the indicated thresholds were changed with p<0.05. **B**, Robust induction of gene expression occurs with low-dose 5-aza. Box-whisker plot of expression levels of genes (1296) changed among groups >1.2-fold and p<0.02 ANOVA, Benjamini Hochberg (BH) corrected indicated a upward shift in gene expression with 5-aza. **C**, Low dose 5-aza induced an early and unique program of gene expression compared to cisplatin. Unsupervised hierarchical cluster analysis of the expression profile of 5-aza and cisplatin altered genes was performed on the 898 genes (rows) altered between the samples (columns) more than 1.5-fold with p value<0.01 ANOVA, BH corrected. Upregulated gene are red, downregulated genes are green. Large overlap in 1 day and 3 day 5-aza treatments and distinct regulation compared to cisplatin is evident. Genes regulated in a similar manner by 5-aza and cisplatin are in brackets.

**Figure 3:**
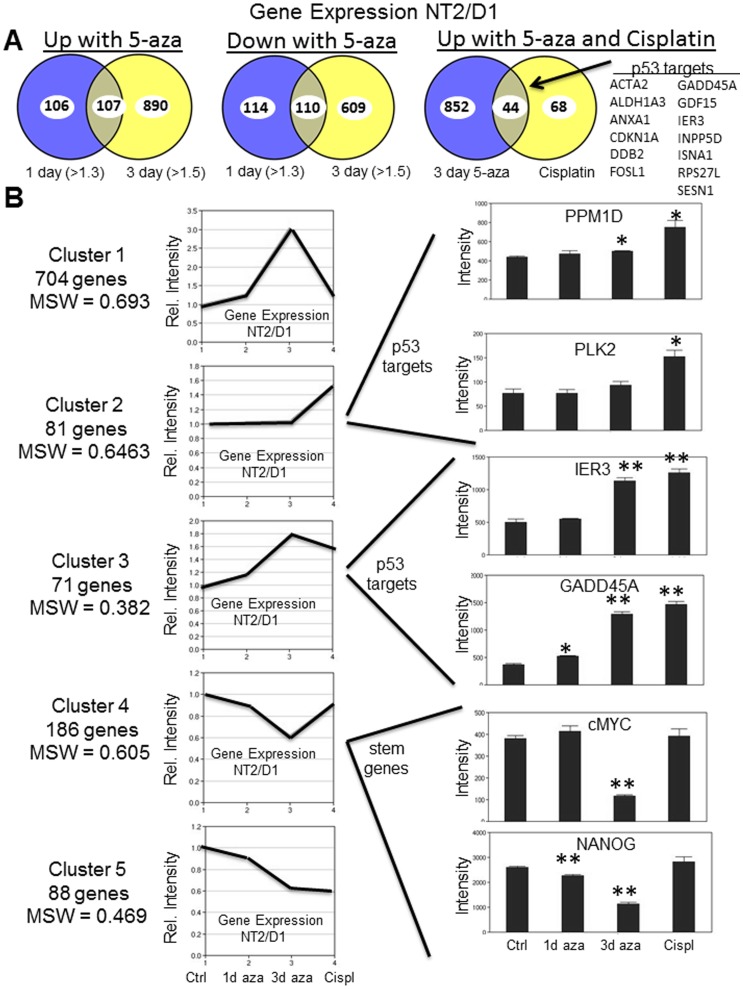
Low-dose 5-aza induces distinct genome-wide activation of p53 target genes and unique repression of pluripotency genes in NT2/D1 cells. **A**, Venn diagrams of expression microarray data from Figure 2 indicating a large overlap in the genes upregulated (left) and down regulated (middle) in NT2/D1 cells with 1 day 5-aza treatment (fold change >1.3) compared to 3 day 5-aza treatment (fold change >1.5). A Venn diagram (right) of microarray data from Figure 2 showing a large degree of overlap in genes upregulated 1.5-fold or greater by both 3 day 5-aza and cisplatin treatments. Genes listed in each Venn diagram are altered with p<0.05. Of the overlap genes, 13 are known p53 target genes in NT2/D1 cells. **B**, Partitioning around mediods (PAM) analysis of 5-aza and cisplatin regulated genes in NT2/D1 cells. The 1130 genes changed 1.5-fold or greater with BH corrected p-value of <0.02 were subjected to PAM analysis as described in Methods. The number of genes in each of the five clusters and the mean silhouette width (MSW) value for each cluster is indicated. Expression intensity values for representative genes in Cluster 2, Cluster 3, and Cluster 4 is provided on the left. Error bars are S.E.M. * = p<0.05; ** = p<0.005 compared to untreated controls.

Array data indicted a robust reprogramming of gene expression after 3 day 5-aza treatment with a bias toward upregulated genes. This is evident in scatter plots and low stringency pairwise statistical analysis (Fig. 2A). Box whisker plots of genes altered 1.2-fold or greater also demonstrates a bias for upregulated genes after 3 day 5-aza treatment that is not apparent in cells treated with cisplatin (Fig. 2B). This finding is consistent with the known demethylating activity of 5-aza to mediate direct induction, as opposed to repression, of gene expression. Compared to the number of genes altered with 3 day 5-aza, very few genes were changed 1.5-fold or greater after 1 day 5-aza and the number of gene changed with cisplatin was substantially fewer compared to 3 day 5-aza (Fig. 2A). Hierarchical cluster analysis was performed on the 898 genes changed more that 1.5-fold between the 4 treatment arms (Fig. 2C). The genes are provided in Table S1. The genes cluster into distinct patterns where a subset of genes are regulated in a similar manner by 5-aza and cisplatin treatment (Fig. 2C, in brackets). However, there are large and prominent clusters of genes that are up or down regulated with only 5-aza and to a much lesser extent with only cisplatin. This pattern suggests that 5-aza and cisplatin share common mechanisms of action but that 5-aza has additional mechanisms not shared by cisplatin. Interestingly, while the number of genes with 1.5-fold changes after 1 day 5-aza is small (35 genes), many of the genes changed after 3 days 5-aza also are similarly regulated after only 1 day 5-aza, albeit with a low-fold threshold (Fig. 2C). Approximately 50% of the genes up or down regulated by 1 day 5-aza above a less stringent threshold of 1.3-fold also were regulated more than 1.5-fold after 3 day 5-aza (Fig. 3A). These results suggest that for a substantial subset of genes altered with 5-aza, expression changes begin early, within 1 day of 5-aza treatment.

### Low-dose 5-aza induces genome-wide activation of p53 target genes and repression of pluripotency genes in NT2/D1 cells

Partitioning around medoids (PAM) analysis was performed on the 1130 genes that changed 1.5-fold or greater between control, 1 day 5-aza, 3 day 5-aza, and cisplatin treatments (Fig. 3B). The genes of each cluster are provided in Table S2. Five distinct clusters again suggest that many of the 5-aza changes begin within 1 day of treatment. The biggest cluster (Cluster 1) represents 704 genes that were primarily upregulated only with 3 day 5-aza. These genes are candidates for mediating the unique hypersensitivity of 5-aza in EC cells. By contrast the 71 genes of Cluster 3 that were co-upregulated by 5-aza and cisplatin were prominently enriched for p53 target genes including IER3, p21 and GADD45A (Fig. 3B). Of the 44 genes upregulated 1.5-fold or greater by both cisplatin and 3 day 5-aza, 13 were previously identified as cisplatin-inducible p53-target genes in our analysis of NT2/D1 cells (Fig. 3A) [25]. Cluster 2 represents genes induced only by cisplatin and were also enriched for p53 targets including PLK2 and PPM1D suggesting that 5-aza induces a subset of the p53 target genes induced by the DNA damaging agent cisplatin. While a set of 88 genes represented by Cluster 5 are repressed by both 5-aza and cisplatin, a larger set of genes in Cluster 4 (186 genes) were only repressed after 5-aza treatment. Interestingly, Cluster 4 is enriched in pluripotency genes including Myc, NANOG and GDF3 suggesting that 5-aza acutely downregulates master regulators of pluripotency in NT2/D1 cells. Expression changes of representative genes for each cluster were confirmed in independent samples by real-time PCR (Fig S1 and data not shown).

Gene Set Enrichment Analysis (GSEA) was performed (Table 1 and Fig S2). Genes upregulated with cisplatin were highly enriched for gene sets corresponding to apoptosis, DNA damage, and p53 target genes (Table 1). The gene set with the highest normalized enrichment score (NES) was from our previous microarray based observation that cisplatin mediates a p53-dominant transcriptional response in NT2/D1 cells [25]. Those genes upregulated with 5-aza are also enriched for gene sets corresponding to apoptosis, DNA damage, and p53 target genes. However, there were gene sets significantly depleted only after 5-aza treatment that are highly expressed in ES cells and representative of core stem cell and pluripotency pathways [6]–[9] (Table 1 and Fig S2). Target genes of the induced pluripotency core stem cell factor, Myc, were also highly repressed by 5-aza in NT2/D1 cells [26], [27] (Table 1). Myc is known to mediate transcriptional programs promoting stem cell renewal. DAVID analysis also indicated that 5-aza represses ES genes and genes with binding sites for pluripotent transcription factors SRY and OCT (Table 1). Additionally, several gene sets comprised of genes previously shown to be induced or repressed by high-dose 5-aza in somatic cancer cells were enriched or depleted after low-dose 5-aza treatment of NT2/D1 cells [28], [29] (Table 1). These genes are distinct from the p53 target and pluripotent gene sets mentioned above. These results suggest that cisplatin and 5-aza share mechanism of toxicity represented by DNA damage inducible p53 target genes but additional mechanisms related to anti-pluripotency and demethylation likely occur with 5-aza. This difference may account for the sensitivity of cisplatin-resistant NT2/D1-R1 cells to 5-aza.

**Table 1:** Gene Sets Enriched with Cisplatin or 5-aza in NT2 cells.

Gene Sets Enriched in NT2+Cisplatin				
Gene Set	Size	NES	p-val	FDR q-val
KERLEY_RESPONSE_TO_CISPLATIN_UP	39	−3.03933	0	0
AMIT_EGF_RESPONSE_480_HELA	161	−2.59991	0	0
KANNAN_TP53_TARGETS_UP	48	−2.5961	0	0
GENTILE_UV_LOW-DOSE-UP	18	−2.5543	0	0
SCHAVOLT_TARGETS_OF_TP53_AND_TP63	16	−2.41143	0	0
DACOSTA_UV_RESPONSE_VIA_ERCC3_UP	307	−2.34113	0	0
GENTILE_UV_LOW-DOSE-UP	18	−2.32132	0	0
INGA_TP53_TARGETS	16	−2.30979	0	0
WEIGEL_OXIDATIVE_STRESS_RESPONSE	30	−2.30105	0	0
CONCANNON_APOPTOSIS_BY_EPOXOMICIN_UP	233	−2.28992	0	0
AMUNDSON_DNA_DAMAGE_RESPONSE_TP53	16	−2.28279	0	0
AMIT_EGF_RESPONSE_120_MCF10A	42	−2.27779	0	0

Gene Sets Enriched in NT2+3 day 5-aza				
Gene Set	Size	NES	p-val	FDR q-val
KERLEY_RESPONSE_TO_CISPLATIN_UP	39	−2.44077	0	0
CONCANNON_APOPTOSIS_BY_EPOXOMICIN_UP	233	−2.21398	0	0
MISSIAGLIA_REGULATED_BY_METHYLATION_UP	99	−2.16867	0	0
MUELLER_METHYLATED_IN_GLIOBLASTOMA	42	−2.09614	0	0
KYNG_DNA_DAMAGE_BY_UV	25	−2.03199	0	9.89E-04
RUGO_UV_RESPONSE	25	−1.96758	0	0.001171
KIM_RESPONSE_TO_TSA_AND_DECITABINE_UP	129	−1.96653	0	0.001156
HAMAI_APOPTOSIS_VIA_TRAIL_DN	125	−1.96154	0	0.001237
HELLER_SILENCED_BY_METHYLATION_UP	248	−1.96044	0	0.001354
INGA_TP53_TARGETS	16	−1.93275	0	0.001516

Gene Sets Depleted in NT2+3 d 5-aza				
Gene Set	Size	NES	p-val	FDR q-val
BENPORATH_ES_1	372	2.698071	0	0
SCHUHMACHER_MYC_TARGETS_UP	69	2.566172	0	0
WONG_EMBRYONIC_STEM_CELL_CORE	331	2.427912	0	0
SCHLOSSER_MYC_TARGETS_REPRESSED_BY_SERUM	158	2.404296	0	0
BENPORATH_ES_2	37	2.395315	0	0
SCHLOSSER_MYC_TARGETS_AND_SERUM_RESPONSE_DN	47	2.282078	0	0
VSE2F_02	173	2.270841	0	0
MUELLER_PLURINET	294	2.269409	0	0
BHATTACHARYA_EMBRYONIC_STEM_CELL	75	2.213246	0	0
PAL_PRMT5_TARGETS_UP	182	2.204928	0	0
MISSIAGLIA_REGULATED_BY_METHYLATION_DN	97	2.171968	0	3.67E-04
CONRAD_STEM_CELL	37	2.16267	0	3.38E-04

DAVID Gene Sets Depleted in NT2+3 d 5-aza (>0.5 fold change)				
Gene Set	p-val	Benjamin		
CGAP_SAGE_QUARTILE Stem cell 3rd	2.30E-38	7.90E-36		
UCSC_TFBS E2F	9.00E-16	7.80E-14		
UCSC_TFBS SRY	1.90E-14	1.10E-12		
UCSC_TFBS OCT	5.20E-10	2.30E-09		

### DNMT3B knockdown alters the low-dose 5-aza response in NT2/D1-R1 cells at a level downstream of p53 induction and DNA damage

**Figure 4:**
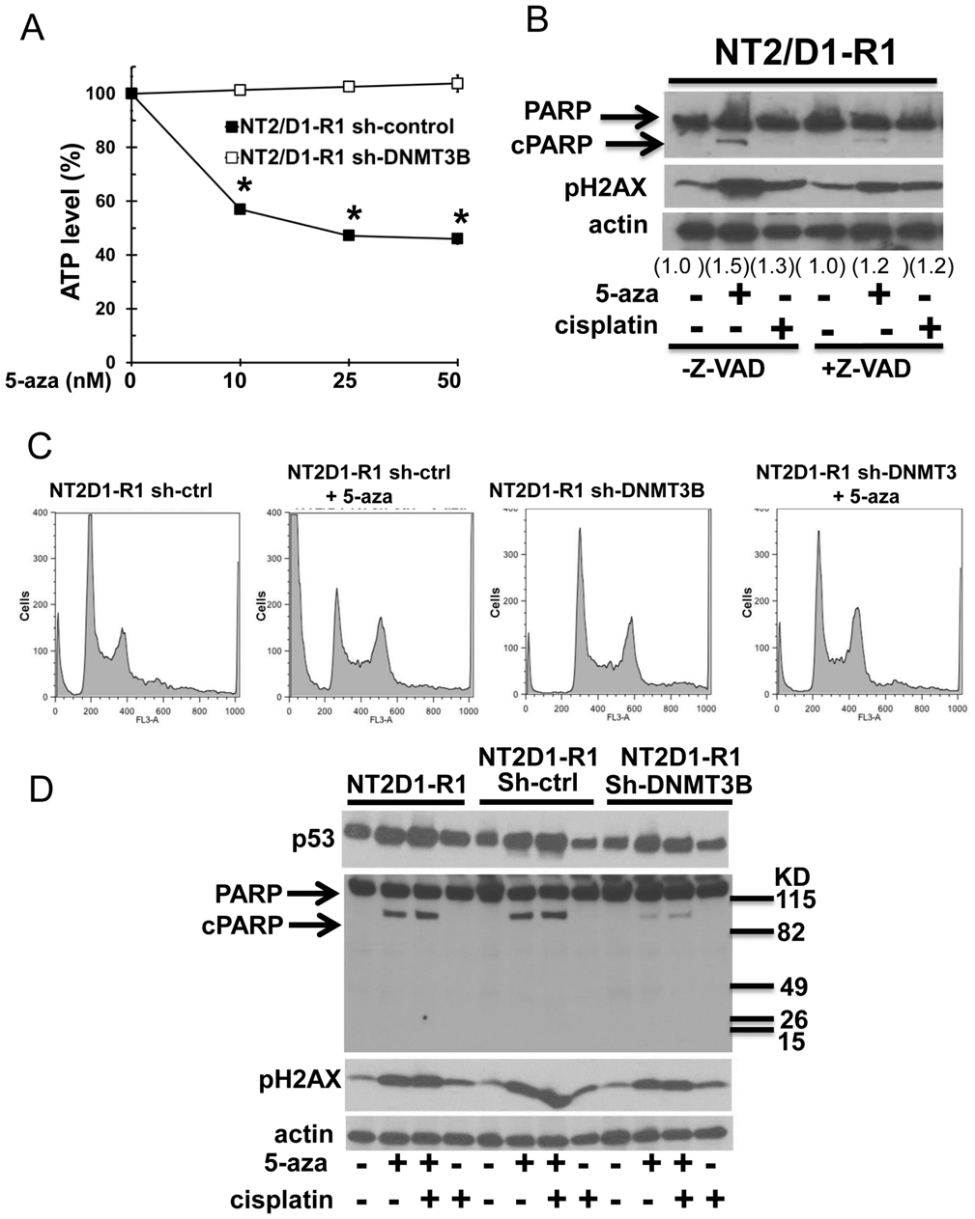
Knockdown of DNMT3B results in resistance to low-dose 5-aza downstream of DNA damage and p53 protein induction. **A**, DNMT3B knockdown in NT2/D1-R1 cells leads to resistance to low-dose 5-aza. Indicated doses of 5-aza were added fresh each day for 3 days to exponentially growing cultures of NT2/D1-R1 lentiviral control cells and NT2/D1-R1 cells stably expressing a lentiviral shDNMT3B construct. Viable cell growth and survival were measured. Data was normalized to no drug treatment. Error bars (within symbols) are standard deviation. * p = <0.05 compared to untreated controls. **B**, Low dose 5-aza induces DNA damage in NT2/D1-R1 cells independent of apoptosis. Cells were pretreated with vehicle or 20 µM Z-VAD-FMK (z-VAD) and treated with 100 nM 5-aza or 2 µM cisplatin for 1 day prior to Western analysis. Accumulation of pH2AX is indicative of double strand breaks. The arrow labeled cPARP indicates cleaved PARP that is indicative of apoptosis. Densitometry of pH2AX normalized to actin is shown. **C**, DNMT3B knockdown in NT2/D1-R1 cells results in resistant to low-dose 5-aza induced cell death and G2 cell cycle arrest. Cells were treated with 10 nM 5-aza for 3 days and then assayed for cell cycle analysis. Substantial cells in subG1 are indicative of cell death. **D**, Knockdown of DNMT3B in NT2/D1-R1 cells inhibits low-dose 5-aza mediated cell death but not p53 protein induction or induction of DNA damage. NT2/D1-R1 cells with no lentivirus and cells treated with sh-control lentvirus and sh-DNMT3B lentivirus were treated with 10 nM 5-aza for 3 days, 1 µM cisplatin for 12 hours or the combination. Western analysis was performed for p53, PARP and pH2AX.

We previously demonstrated that knockdown of DNMT3B confers substantial resistance to 5-aza in NT2/D1 and NT2/D1-R1 cells [21]. Since 5-aza resistance to DNMT3B knockdown is particularly dramatic in cisplatin-resistant NT2/D1-R1 cells, these cells were used to study the dependence of DNMT3B on 5-aza treatment of EC cells. Knockdown of DNMT3B in NT2/D1-R1 cells results in extensive resistance to low-dose 3 day 5-aza treatment compared to sh-control cells (Fig. 4A). As in NT2/D1 cells (Fig. 1D, Fig. 1E) 5-aza treatment of NT2/D1-R1 cells induced cell death as determined by PARP cleavage and sub G1 DNA content with G2 arrest (Fig. 4B and Fig. 4C). Three day 5-aza treatment also induced DNA damage as assessed by induction of phosphorylated H2AX (pH2AX). As expected, cisplatin does not induce PARP cleavage in cisplatin resistant NT2/D1-R1 cells but can induce pH2AX, strongly suggesting that cisplatin resistance is downstream of effective DNA damage induction (Fig. 4B). Importantly, 5-aza (100 nM) mediated pH2AX activation also occurs in the presence of the caspase 3 inhibitor (Z-VAD-FMK) at concentrations that inhibit PARP cleavage (Fig. 4B). Further pH2AX accumulation begins in NT2/D1-R1 cells within 1 day of low dose (10 nM) 5-aza treatment while cleaved PARP can only be seen after 2 days with this dose of 5-aza (Fig S3). These results suggest that the DNA damage mediated by low-dose 5-aza treatment of NT2/D1-R1 cells is a primary event.

Knockdown of DNMT3B in NT2/D1-R1 cells results in a substantial decrease in PARP cleavage compared to wild-type and sh-control NT2/D1-R1 cells. However, activation of H2AX and induction of p53 is similar in sh-control and sh-DNMT3B cells (Fig. 4D). Taken together, the data indicates that low-dose 5-aza is sufficient to cause DNA damage in NT2/D1 and NT2/D1-R1 cells. However, DNMT3B knockdown does not appear to alter 5-aza mediated DNA damage. Hence resistance to 5-aza seen upon DNMT3B knockdown appears to be due to a defect downstream of the induction of DNA damage. The data also suggests that induction of DNA damage in and of itself is insufficient to account the 5-aza hypersensitivity of NT2/D1-R1 cells.

### DNMT3B knockdown opposes low-dose 5-aza genome-wide activation of p53 target genes and repression of pluripotency genes in NT2/D1-R1 cells

**Figure 5:**
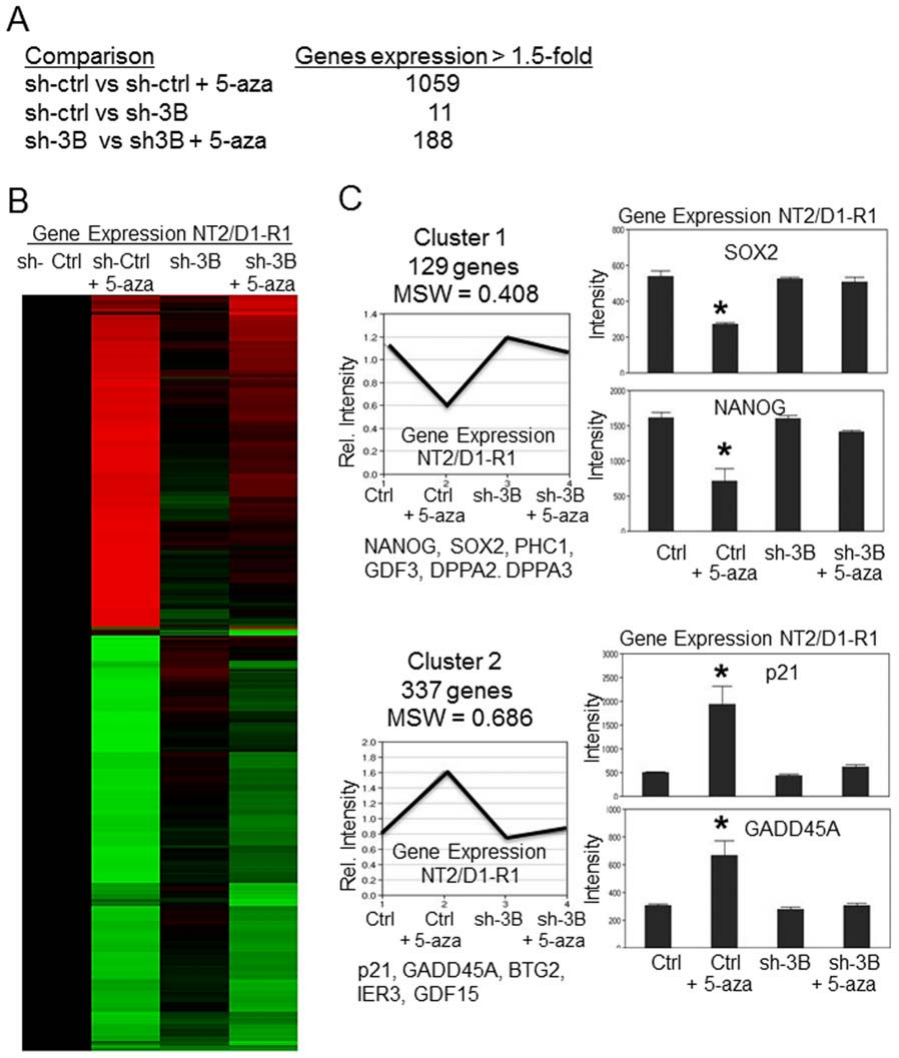
DNMT3B knockdown inhibits low-dose 5-aza mediated genome-wide activation of p53 target genes and repression of pluripotency genes. **A**, Summary of results from expression microarray data of NT2/D1-R1 sh-control cells (sh-ctrl) and NT2/D1-R1 sh-DNMT3B cells (sh3B) untreated or treated for 3 days with 10 nM 5-aza. Knockdown of DNMT3B greatly reduced the number of genes altered with 5-aza treatment while DNMT3B knockdown alone results in minimal expression changes. The number of genes altered was based on the average of 3 biological replicates with 1.5-fold or greater change and a p value<0.01. **B**, Unsupervised hierarchical cluster analysis of the expression data in (A) was performed on the 541 genes altered more than 1.8-fold with p value<0.01 ANOVA BH corrected, between NT2/D1-R1 sh-control cells (sh-ctrl) and NT2/D1-R1 sh-DNMT3B cells (sh3B) untreated or treated for 3 days with 10 nM 5-aza. Upregulated gene are red, downregulated genes are green. C, Partitioning around mediods (PAM) analysis of 1169 genes changed 1.5-fold or greater with BH corrected p-value of <0.01. Cluster 1 and Cluster 2 of 6 total clusters are shown. All clusters are provided in supplemental Fig S4. The number of genes in the clusters and the mean silhouette width (MSW) value is indicated. Expression intensity values for representative genes in Cluster 1 (pluripotent genes) and Cluster 2 (p53 target genes) is provided on the right and additional prominent members are provided below each cluster. Error bars are S.E.M. * = p<0.05 compared to untreated control.

Genome-wide expression analysis was performed on NT2/D1-R1 control and DNMT3B knockdown cells treated for 3 days with low-dose 5-aza. There was a large degree of overlap (approximately 20%) in 5-aza responsive genes in NT2/D1-R1 (NT2/D1-R1 vs. NT2/D1-R1+5-aza) compared to NT2/D1 cells (NT2/D1 vs. NT2/D1+5-aza) and those genes upregulated by 5-aza in NT2/D1-R1 cells were again associated with DNA damage and p53 while those genes repressed by 5-aza were associated with stemness and pluripotency (data not shown). There were very few genes changed basally due to DNMT3B knockdown alone in NT2/D1-R1 cells and little correlation was observed between these few genes and the genes altered by 5-aza treatment of NT2/D1-R1 cells (one shared gene, ZCCHC12, Fig. 5A and Fig. 5B). These data indicate that DNMT3B knockdown alone is insufficient to allow re-expression of DNA methylated genes in NT2/D1-R1 cells. However, knockdown of DNMT3B substantially suppresses 5-aza mediated gene expression changes (Fig. 5A and Fig. 5B).

PAM analysis was performed on 1169 genes changed 1.5-fold or greater between sh-control, sh-control+5-aza, sh-DNMT3B, and sh-DNMT3B+5-aza groups (Fig. 5C). Of the 6 clusters identified, Cluster 1 and Cluster 2 are particularly informative (see Fig S4 for all clusters). Cluster 1 represents 129 genes that were downregulated by 5-aza in control cells but not by 5-aza in DNMT3B knockdown cells. These genes are enriched for pluripotency genes including NANOG, SOX2, PHC1, GDF3, DPPA2 and DPPA3 (Stella). Cluster 2 represents 337 genes that are induced by 5-aza only in control cells and not in sh-DNMT3B cells. These include p53 target genes p21, GADD45A, BTG2, IER3 and GDF15. Lists of the genes represented in Fig. 5B and Fig. 5C are provided in Tables S3 and S4. Expression changes of representative genes for Cluster 1 and Cluster 2 were confirmed in independent samples by real-time PCR (Fig S1 and data not shown).

GSEA also strongly indicated that DNMT3B knockdown impeded 5-aza repression of pluripotency genes and 5-aza induction of p53 target and apoptotic genes in NT2/D1-R1 cells (Fig S5). Gene sets depleted in 5-aza treated control cells compared to 5-aza treated DNMT3B cells (i.e. no longer repressed by 5-aza in DNMT3B knockdown cells) include genes sets for ES genes, OCT4 targets, and genes on chromosome 12p13 which is a hot-spot region for pluripotency in ES and EC cells [30], [31] (Fig S5). Gene sets enriched in 5-aza treated control cells compared to 5-aza treated DNMT3B cells (i.e. no longer induced by 5-aza in DNMT3B knockdown cells) include gene sets for apoptotic and p53 target genes. Interestingly, a study by Missiaglia *et al.*, assessed global gene expression changes in pancreatic cancer cells 6 days after a 24-hour treatment of 2 µM 5-aza [28]. GSEA indicated that these genes were no longer regulated to the same extent by low-dose 5-aza in DNMT3B knockdown cells (Fig S5). The genes are distinct from the pluripotency and p53 target genes discussed above.

### Genome-wide promoter methylation after low-dose 5-aza treatment of NT2/D1-R1 cells

**Figure 6:**
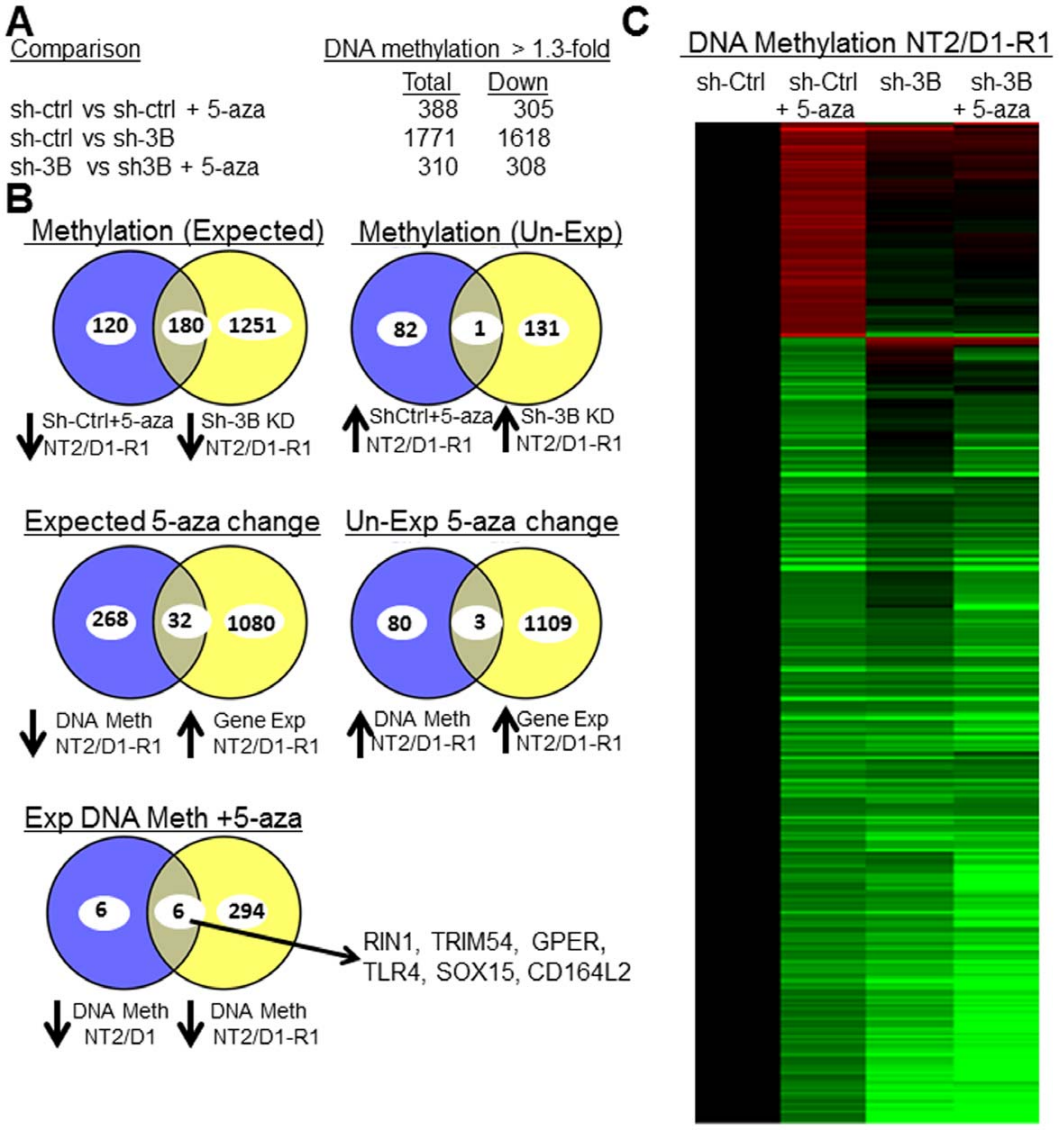
Low-dose 5-aza and DNMT3B knockdown alters genome-wide promoter demethylation in NT2/D1-R1 cells. **A**, Summary of results from Illumina 27K beadchip DNA promoter methylation analysis of NT2/D1-R1 sh-control cells (sh-ctrl) and NT2/D1-R1 sh-DNMT3B cells (sh3B) untreated or treated for 3 days with 10 nM 5-aza. The number of promoter methylation alterations was based on the average of 3 replicates with 1.3-fold or greater change and a p value<0.05. **B**, Venn diagrams depicting degree of overlap in genes altered in expression or DNA promoter methylation in NT2/D1-R1 cells due to low dose 5-aza or DNMT3B knockdown. **Top**, High degree of overlap in genes in NT2/D1-R1 cells demethylated with low dose 5-aza and demethylated with DNMT3B knockdown (left). Little overlap in genes in NT2/D1-R1 cells undergoing increased DNA methylation with low dose 5-aza and increased DNA methylation with DNMT3B knockdown (right). The numbers represent methylation changes of 1.3-fold or greater with p<0.05. **Middle**, A moderate degree of overlap in genes in NT2/D1-R1 cells that underwent decreased DNA methylation and increased gene expression with 5-aza (left) and little overlap in genes in NT2/D1-R1 cells that underwent increased DNA methylation and increased gene expression with 5-aza. The numbers represent methylation changes of 1.3-fold or greater with p<0.05 and expression changes of 1.5-fold or greater with p<0.01. **Bottom**, A large degree of overlap in genes demethylated after 5-aza treatment of NT2/D1 and NT2/D1-R1 cells. The six overlapping genes are shown. The numbers represent methylation changes of 1.3-fold or greater with p<0.05. **C**, Unsupervised hierarchical clustering of the promoter DNA methylation of the 4 treatment arm values among just the 388 genes changed 1.3-fold or greater, p value<0.05 with 5-aza in the control cells depicting the large degree of overlap in genes undergoing demethylation with 5-aza and DNMT3B knockdown. Increased methylation = red, decreased methylation = green.

Genome-wide effects of low-dose 5-aza and DNMT3B knockdown on promoter methylation was assessed (Table S5). In contrast to genome-wide expression analysis where few genes in NT2/D1-R1 cells were altered by DNMT3B knockdown alone (Fig. 5A), many gene promoters showed DNA methylation changes upon DNMT3B knockdown and the great majority of these showed decreased methylation (Fig. 6A). This indicates that knockdown of DNMT3B alone is sufficient for NT2/D1-R1 cells to undergo wide-spread promoter DNA hypomethylation but is not sufficient for gene re-expression. The results also imply promoter DNA hypomethylation alone cannot fully account for the robust effects of 5-aza on gene expression in NT2/D1-R1 cells.

Low-dose 3 day 5-aza treatment of control NT2/D1-R1 cells altered the promoter methylation of a smaller set of genes compared to DNMT3B knockdown and again the majority of genes had decreased methylation (Fig. 6A). Strikingly, approximately 60% of the genes with decreased promoter methylation with 5-aza also demonstrated decreased methylation with DNMT3B knockdown (Fig. 6B top). This can be seen by hierarchical clustering of the 4 treatment arm values for just the 388 genes with significant methylation changes with 5-aza in control cells (Fig. 6C). As expected, there was little overlap in genes with increased promoter methylation with 5-aza treatment in control cells and DNMT3B knockdown (Fig. 6B top, Fig. 6C). Approximately 10% of genes with decreased methylation with 5-aza also showed increased gene expression after 5-aza treatment (Fig. 6B middle). In contrast, approximately 4% of the genes with increased methylation showed an unexpected increase in gene expression with 5-aza (Fig. 6B middle).

**Figure 7:**
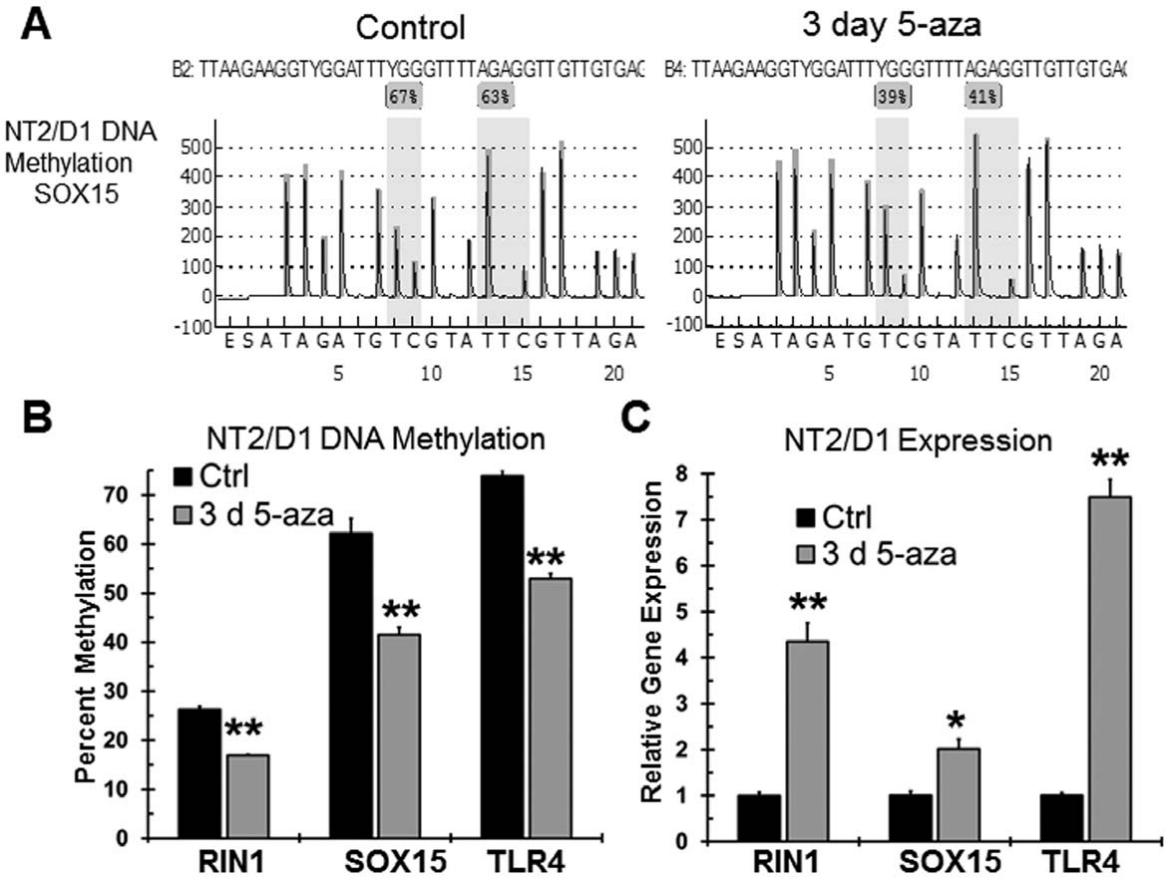
Confirmation of promoter DNA demethylation with low-dose 5-aza in NT2/D1 cells. **A**, Representative bisulfite pyrosequencing tracing from NT2/D1 cells treated with vehicle control or 10 nM 5-aza for 3 days in the promoter region of SOX15. Shaded areas are CpG sites. **B**, Decrease in DNA methylation of the RIN1, SOX15 and TLR4 promoter with 3 day 10 nM 5-aza treatment of NT2/D1 cells as determined by bisulfite pyrosequencing. Average of biological triplicate determinations. Error bars are standard deviation. ** = p<0.005. SOX15 values represent the average methylation value across two CpG sites. RIN1 and TRL4 values represent the average methylation across three CpG sites. **C**, Increased gene expression of RIN1, SOX15 and TLR4 with 3 day 10 nM 5-aza treatment of NT2/D1 cells as determined by real-time PCR. Average of biological triplicate determinations. Error bars are standard deviation. * = p<0.05; ** = p<0.005.

Global DNA promoter methylation analysis was also performed in NT2/D1 cells after 3 day low-dose 5-aza treatment (data not shown). For unclear reasons, methylation changes were less robust for the NT2/D1 experiments with only 12 genes having significantly decreased promoter methylation. However, 6 of the 12 genes with decreased methylation in NT2/D1 cells also had decreased methylation in NT2/D1-R1 cells with 5-aza (Fig. 6B bottom). These genes are RIN1, SOX15, TLR4, GPER, TRIM54, and CD164L2. Importantly, bisulfite pryrosequencing and real-time PCR of independent samples confirmed that three of the genes, RIN1, SOX15 and TLR4 underwent decreased promoter methylation and increased expression in NT2/D1 cells treated with 3 day low-dose 5-aza (Fig. 7).

## Discussion

**Figure 8:**
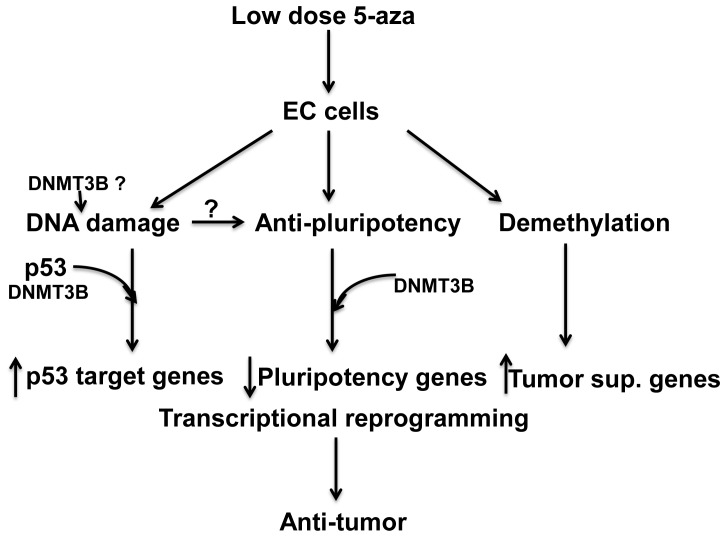
Model of mechanisms of low-dose 5-aza hypersensitivity in EC cells. Low dose 5-aza effects on EC cells are multifactorial and include induction of DNA damage, p53 activation, DNA promoter demethylation, and repression of pluripotency genes. Proposed DNMT3B-dependent effects based largely on microarray analysis is indicated along with speculative role of DNMT3B in altering composition of DNA adducts. Prior direct repression of pluripotency factors by DNA damage is not supported by the current study.

In prior work we established that EC cells are exquisitely sensitive to low nanomolar doses of 5-aza and that this hypersensitivity is partially dependent on high levels of DNMT3B. The current study utilizes genome-wide transcriptional and promoter methylation analyses to discover that 5-aza not only exerts its biological effects through DNA demethylation and gene re-expression but also by an unexpected repression of pluripotency in NT2/D1 cells. Further, low dose 5-aza mediates DNA damage as assessed by accumulation of pH2AX that is associated with a robust induction of p53 target genes. Interestingly, the transcriptional reprogramming with 5-aza in NT2/D1-R1 cells appears largely dependent on high levels of DNMT3B while the accumulation of pH2AX and p53 does not. The relationship between DNA damage, p53 target gene activation, DNA demethylation, and repression of pluripotency in response to low-dose 5-aza in NT2/D1-R1 cells is depicted in Figure 8.

The exact anticancer mechanism of 5-aza is controversial and most studies have used somatic cells at doses substantially higher than used in the current study. There is evidence that incorporation of 5-aza in DNA results in adduct formation with DNMTs leading to sequestration-mediated hypomethylation or a trigger for a DNA-damage response [18]–[20], [32], [33]. Doses of 5-aza as low as 10 nM are sufficient to induce DNA damage and apoptosis in NT2/D1 cells as monitored by pH2AX (Fig. 1) and this is associated with the induction of a classic p53 target gene signature coupled with transcriptional repression of core pluripotency genes (Fig. 3). Interestingly, the transcriptional effects commence even after only 1 day of 5-aza treatment suggesting that they are proximal to the actions of 5-aza on NT2/D1 cells (Fig. 2). The data also suggests that either one cell cycle is sufficient to meet threshold 5-aza incorporation for demethylation/DNA damage responses or that 5-aza can have additional effects on EC cells independent of DNA incorporation.

We and others have shown that EC cells undergo hyperactive activation of pro-apoptotic p53 target genes in response to cisplatin, suggesting that TGCTs responsiveness to DNA damaging agents may relate to a unique cellular context during p53 activation [25], [34]–[36]. Notably, recent evidence suggests that p53 is a barrier for induced pluripotency of somatic cells and has antistemness and prodifferentiation functions in ES and EC cells [37]–[40]. It has also been suggested that p53 can directly repress pluripotency genes including NANOG, OCT4 and GDF3 after treatment with DNA damaging agents [41], [42]. However, we failed to see downregulation of pluripotency genes with cisplatin under the conditions employed here and 5-aza repression of pluripotency genes was not affected by shRNA knockdown of p53 (data not shown). Interestingly, 5-aza only induced a subset of the p53 target genes that were induced with cisplatin, further suggesting that 5-aza and cisplatin may activate p53 in distinct manners, which for 5-aza may include mechanisms apart from DNA damage.

Doses of 5-aza as low as 10 nM led to global hypomethylation of LINE-1 repetitive elements and a decrease in promoter methylation in NT2/D1 and NT2/D1-R1 cells (Fig. 1, Fig. 6, and Fig. 7). The promoter hypomethylation was in both CpG islands and non CpG island promoters. The genes induced only by 5-aza (Cluster 1, Fig. 3) may be induced by a demethylation mechanism and are likely important in mediating the acute hypersensitivity of 5-aza in NT2/D1 cells. There was also a substantial degree of overlap between genes with decreased promoter DNA methylation and induced expression with low-dose 5-aza, suggesting effective re-expression through DNA demethylation is occurring (Fig. 6B). RIN1, TLR4 and SOX15 are novel candidate biomarkers and tumors suppressor genes in TGCTs (Fig. 7). In addition, GSEA analysis suggests that a subset of genes altered with high-dose 5-aza in other tumor types is also altered with low-dose 5-aza in NT2/D1 cells, further supporting demethylation as a participating mechanism for the hypersensitivity of NT2/D1 cells to 5-aza.

Recent analysis of the methylome of ES and induced pluripotent stem (iPS) cells supports a unique pattern of DNA methylation in ES cells that suggests potential additional mechanisms responsible for the hypersensitivity of EC cells to 5-aza compared to somatic cells [12]–[14], [43]–[45]. A potential mechanism for acute low-dose 5-aza toxicity in EC cells is increased genomic instability due to demethylation of centromeric and pericentromeric satellite repeats [46]. Recently, pluripotent cells have been exclusively shown to possess high non-CpG methylation in gene bodies that correlates with the expression and specificity of DNMT3B [13], [47]. It would be of interest to investigate whether demethylation of non-CpGs plays a role in 5-aza response in EC and in a boarder sense whether the unique methylome of the pluripotent genome sensitizes cells to 5-aza.

The use of 5-aza in the treatment of myelodysplastic syndrome and recent trials in lung cancer suggest low-dose 5-aza treatment mediates delayed and long-term anticancer responses [22], [23]. A possible implication of these findings is that low-dose 5-aza may preferentially target cancer-initiating or stem-like cells and that the prolonged time to response in patients might involve progressive exhaustion of discrete cell populations. An elegant recent report from Tsai and colleagues used *in vivo* and *in vitro* models to demonstrate that 3 day 10 nM 5-aza treatments did not elicit DNA damage or acute toxicity in a variety of solid tumors but did mediate delayed toxicity associated with depletion of tumor initiating cells [24]. It is tempting to speculate that rare malignant stem-like cells in somatic solid tumors may undergo acute toxicity similar to EC cells, and by the mechanisms outlined above (Fig. 8), to account for the delayed responses to 5-aza in bulk somatic solid tumors.

Other reports have also seen that knockdown of DNMT expression in ES cells results in decreased sensitivity to 5-aza [19], [48]. Surprisingly, a similar level of DNA damage with 5-aza, as monitored by pH2AX and p53 induction, was seen in our control and DNMT3B knockdown cells (Fig. 4). However, there is a dramatic decrease in 5-aza mediated repression of pluripotency genes and 5-aza induction of p53 target genes with DNMT3B knockdown (Fig. 5). These results support a cause-and-effect relationship between 5-aza gene expression alterations in NT2/D1-R1 cells and acute toxicity and suggest that the role of DNMT3B in 5-aza toxicity is at a level downstream of induction of DNA damage. One explanation for the apparent paradoxical effect of DNMT3B knockdown on 5-aza mediated gene expression and survival of NT2/D1-R1 cells is that in the absence of DNMT3B DNA adducts formed with 5-aza may be qualitatively or quantitatively altered in a manner that is insensitive to discrimination by pH2AX staining. An example would be differential recruitment of chromatin modifying proteins. Perhaps there is also redistribution of DNA-adducts and DNA damage in DNMT3B knockdown cells that is not detected by total pH2AX staining that originates from the unique localization and high levels of DNMT3B in EC cells [6]–[8], [11], [49], [50]. There is extensive evidence that different DNMTs are differentially recruited to DNA and participate in DNMT isoform-specific protein interactions to exert scaffolding functions for chromatin dynamics and regulation [51]–[55]. DNMT3B- and pH2AX-specific chromatin immunoprecipitation and co-immunoprecipitation assays may help resolve the apparent paradoxical role of DNMT3B in the 5-aza response of NT2/D1 cells.

Interestingly, although few genes were changed in expression by DNMT3B knockdown alone (Fig. 5), there was substantial decreases in gene promoter methylation with DNMT3B knockdown in NT2/D1-R1 cells (Fig. 6) and these promoter demethylations had a large degree of overlap with demethylation induced with 5-aza (Fig. 6B). This data suggests that DNA demethylation with low-dose 5-aza in NT2/D1-R1 cells does not fully explain its acute toxicity and effects on gene expression.

TGCT-derived pluripotent EC cells, even those resistant to cisplatin, are hypersensitive to low-dose 5-aza compared to solid somatic tumors cells [21]. We have shown in NT2/D1 and NT2/D1-R1 cells that this acute, low-dose sensitivity to 5-aza is likely mediated through a multifactorial mechanism involving the combined activation of p53 targets, repression of pluripotency genes, and activation of genes repressed by DNA methylation. Low-dose 5-aza therapy may be a general strategy to treat those tumors that are sustained by cells with embryonic stem-like properties. Studies on pluripotent EC cells may have important biological and clinical relevance based on the growing appreciation that cancer stem or initiating cells share gene expression programs with pluripotent cells and the recent findings that low-dose 5-aza may target tumor initiating cells in solid tumors.

## Materials and Methods

### Cell proliferation and cell cycle analysis

Cell were cultured in DMEM media (Gibco) with 10% FBS. 5-aza (Sigma) was added fresh each day except where indicated. NT2/D1 cells were obtained through ATCC. The derivation of the NT2/D1 resistant cell line NT2/D1-R1 was described previously [40]. Lentiviral control cells and the stable shRNA DNMT3B knockdown cell line (NT2/D1-R1-sh84) were described previously [21]. DNMT3B knockdown was greater than 90% by Western analysis [21]. Cell proliferation and survival was assessed with the Cell-Titre Glo assay (Promega). Cell cycle analysis with propidium iodine was previously described [25]. All other drugs and chemicals were purchased from Sigma.

### Western analysis and Real-time PCR

SYBR green-based real-time PCR (Applied Biosystems) was employed using the ddCT method normalized to GAPDH. Primer sequences are listed in Table S6. For Western analysis cells were lysed in radioimmune precipitation buffer and separated by SDS-PAGE. Antibodies to actin (sc01615, Santa Cruz), 139-H2AX (Cell Signaling), PARP (c-2-10, Biomol International) and p53 (DO-1, Santa Cruz) were used.

### Gene expression microarray analysis

RNA was extracted with Trizol reagent and quality control was performed with the Agilent Bioanalyser. Biotinylated cRNA was prepared with the Ambion MessageAmp kit for Illumina arrays. Expression analysis was performed on the Illumina HumanHT-12 v3 or Illumina HumanHT-12 v4 bead chip arrays and scanned on the BeadArray Reader (Illumia) according to manufacturer's instructions. Raw data was normalized (quantile) with Genome Studio software (Illumina). Data was imported to GeneSifter (vizX labs) for pairwise and ANOVA statistical analyses. Hierarchal clustering was performed using a correlation metric for similarity and average linkage clustering. In all cases the average of biological triplicate values for each treatment group were used for clustering analysis. Partitioning around mediods (PAM) analysis was performed in GeneSifter using a correlation metric for similarity. The PAM algorithm partitions a dataset of n objects into a number of k clusters. The algorithm works with a matrix of dissimilarity, where its goal is to minimize the overall dissimilarity between the genes of each cluster. The number of clusters was chosen empirically to obtain the best mean silhouette width (MSW), a statistic that measures how well each member of the cluster conforms to the mean pattern. GSEA software was downloaded from the Broad website (http://www.broadinstitute.org/gsea/index.jsp). The number of permutations were 1,000 and the permutation type was gene_set.

### Genome-wide methylation analysis

Genomic DNA was extracted and purified using the Qiagen DNeasy Kit according to standard instructions. Bisulphite converted DNA was amplified, fragmented and hybridized to the Illumina Infinium Human Methylation27 Beadchip using standard Illumina protocols. DNA methylation analysis was performed in biological triplicate and the BeadChip was scanned on the BeadArray Reader (Illumina), according to the manufacturer's instructions. Raw data was converted to average beta values with Genome Studio software (Illumina). The HumanMethylation27 BeadChip assays 27,578 CpGs covering more than 14,000 genes, mostly from promoter regions. The arc-sine square root of the average-beta values was imported to GeneSifter (vizX labs) for pairwise and ANOVA statistical analyses. Hierarchical clustering was performed as stated above. HumanMethylation27 BeadChip data and gene expression microarray data have been submitted to the NCBI GEO database.

### LINE and promoter specific pyrosequencing

Genomic DNA was isolated with the QIAMP DNA mini kit (Qiagen) and bisulfite converted with the EZ DNA methylation kit (Zymo Research). DNA was amplified with HotStarTaq plus DNA polymerase (Qiagen). The LINE-1 pyrosequencing assay averages across 4 CpG sites and has been described previously [56]. Pryrosequencing assays for RIN1, SOX15, and TLR4 were designed with PyroMark assay design software (Qiagen) to sequence across corresponding probes cg0599842, cg02515422 and cg13098960 on the HumanMethylation27 BeadChip. Primers sequences for pryrosequencing of RIN1, SOX15 and TLR4 are listed in Table S6.

## Supporting Information

Figure S1 **RT-PCR confirmation of 5-aza dependent gene regulation in NT2/D1, NT2/D1-R1, and DNMT3B knockdown (sh-3B) cells.**
(TIF)

Figure S2 **Gene set enrichment analysis (GSEA) of NT2/D1 cells.** A) Gene sets upregulated with cisplatin, B) Gene sets upregulated with 5-aza, C) Gene sets downregulated with 5-aza.
(TIF)

Figure S3 **Low dose 5-aza induces early DNA damage.** Low dose 5-aza (10 nM) induces pH2AX within 24 hours, but two days of treatment is required for induction of cleaved PARP indicative of apoptosis.
(TIF)

Figure S4 **Partitioning around mediods (PAM) analysis of 5-aza regulated genes in control NT2/D1-R1 and DNMT3B knockdown (sh-3B) cells.**
(TIF)

Figure S5 **DNMT3B knockdown inhibits 5-aza mediated gene expression as determined by GSEA.** Top, Gene sets depleted in 5-aza treated control NT2/D1-R1 cells compared to 5-aza treated DNMT3B KD cells (no longer repressed by 5-aza in DNMT3B KD cells). Bottom, Gene sets enriched in 5-aza treated control cells compared to 5-aza treated DNMT3B KD cells (no longer induced by 5-aza in DNMT3B KD cells).
(TIF)

Table S1 **Genes used for cluster analysis in** **Figure 2C** **.** The 898 gene altered >1.5-fold, p<0.01 ANOVA, Benjamini and Hochberg corrected with either low dose 5-aza or cisplatin in NT2/D1 cells.
(TXT)

Table S2 **Genes clusters for the PAM analysis in** **Figure 3B** **.** Clusters of the 1130 genes altered >1.5-fold, p<0.02 ANOVA, Benjamini and Hochberg corrected with low dose 5-aza or cisplatin in NT2/D1 cells.
(TXT)

Table S3 **Genes used for cluster analysis in** **Figure 5B** **.** The 541 genes altered >1.8-fold, p<0.01 ANOVA, Benjamini and Hochberg corrected between NT2/D1-R1 control or DNMT3B knockdown cells untreated or treated with low dose 5-aza.
(TXT)

Table S4 **Genes clusters for the PAM analysis of** **Figure 5C** **and Figure S3.** Clusters of the 1169 genes altered >1.5-fold, p<0.01 ANOVA Benjamini and Hochberg corrected between NT2/D1-R1 control or DNMT3B knockdown cells untreated or treated with low dose 5-aza.
(TXT)

Table S5 **Genes with DNA methylation changes with 5-aza or DNMT3B knockdown in NT2/D1-R1 cells altered >1.2-fold, p<0.01 ANOVA Benjamini and Hochberg corrected.**
(TXT)

Table S6 **Primers used in this study.**
(XLSX)
