# Supplementary figures and images for: Acute Hypersensitivity of Pluripotent Testicular Cancer-Derived Embryonal Carcinoma to Low-Dose 5-Aza Deoxycytidine Is Associated with Global DNA Damage-Associated p53 Activation, Anti-Pluripotency and DNA Demethylation
Source: PLoS One. 2012 Dec 27;7(12):e53003. doi: 10.1371/journal.pone.0053003 (PMC3531428; doi:10.1371/journal.pone.0053003)

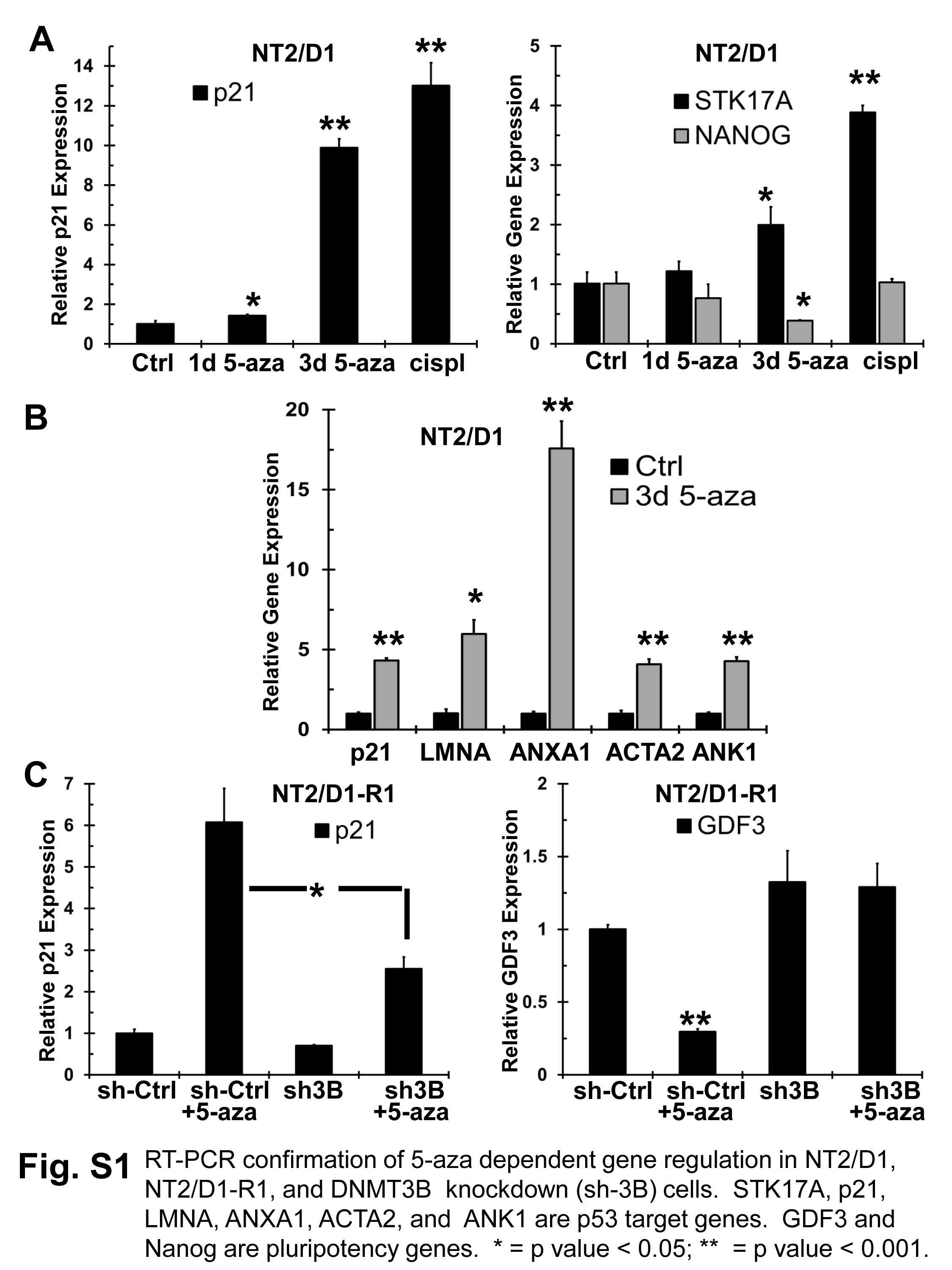

Supplement: Figure S1 — RT-PCR confirmation of 5-aza dependent gene regulation in NT2/D1, NT2/D1-R1, and DNMT3B knockdown (sh-3B) cells. (TIF) [file pone.0053003.s001.tif]

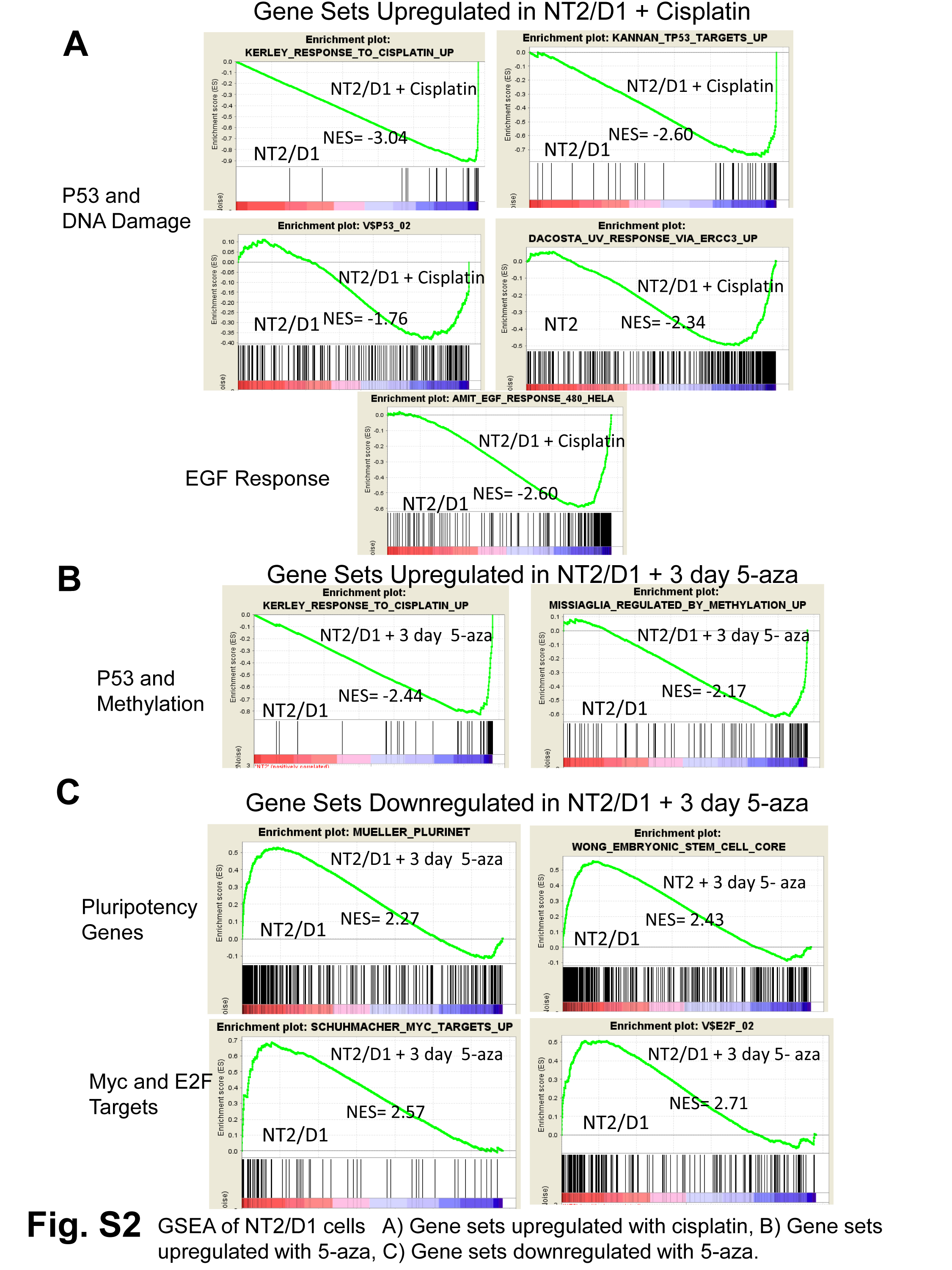

Supplement: Figure S2 — Gene set enrichment analysis (GSEA) of NT2/D1 cells. A) Gene sets upregulated with cisplatin, B) Gene sets upregulated with 5-aza, C) Gene sets downregulated with 5-aza. (TIF) [file pone.0053003.s002.tif]

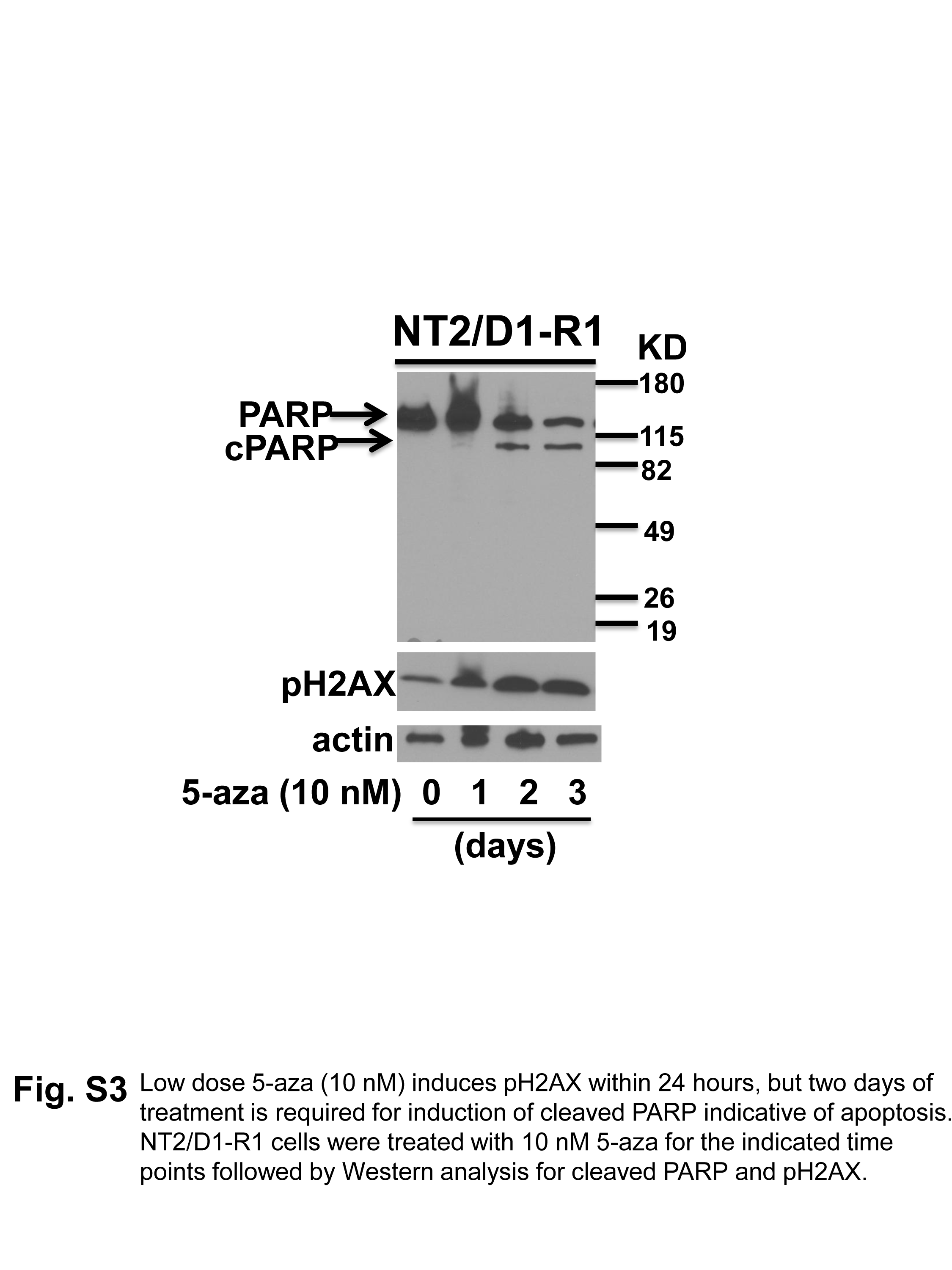

Supplement: Figure S3 — Low dose 5-aza induces early DNA damage. Low dose 5-aza (10 nM) induces pH2AX within 24 hours, but two days of treatment is required for induction of cleaved PARP indicative of apoptosis. (TIF) [file pone.0053003.s003.tif]

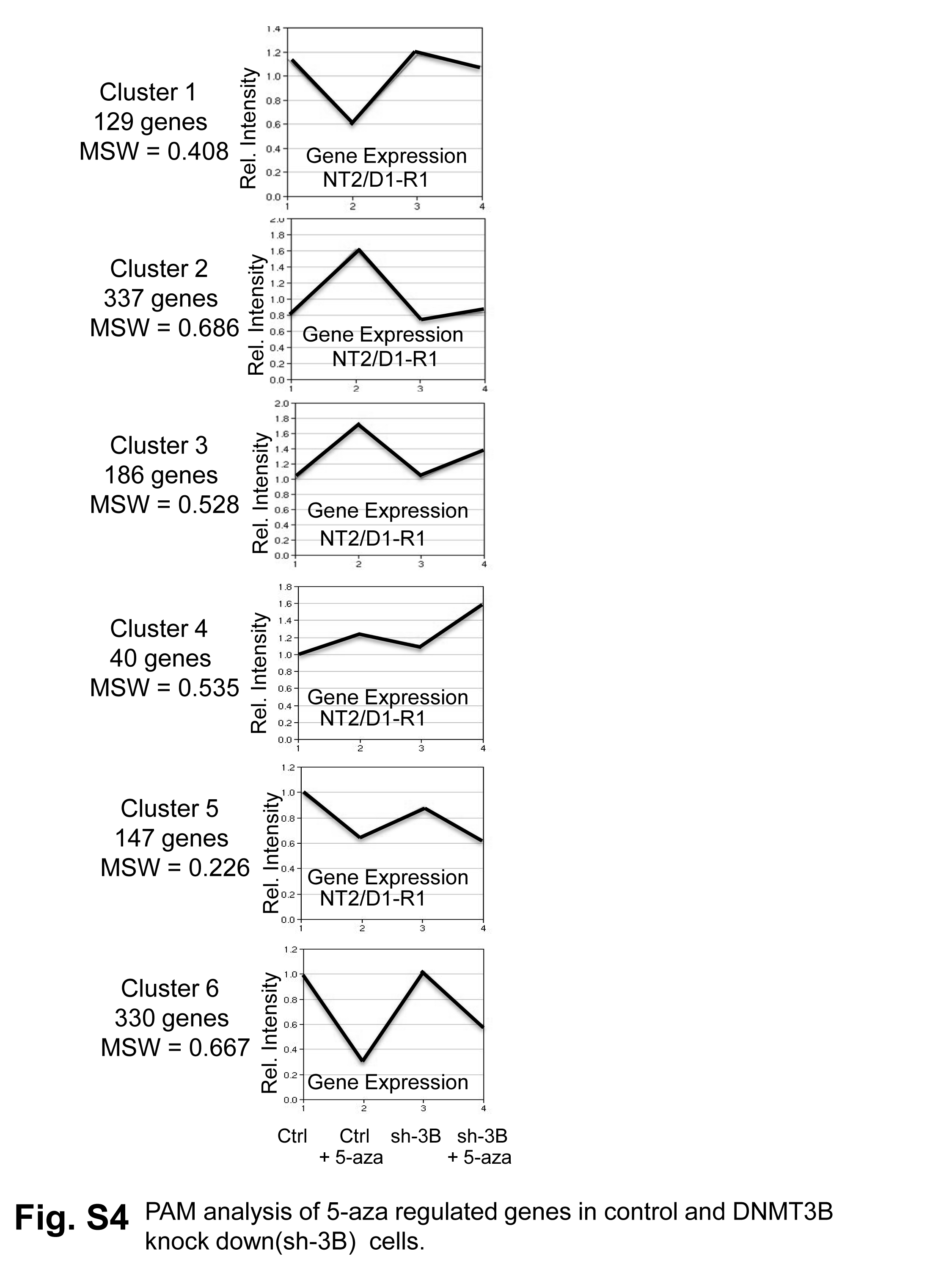

Supplement: Figure S4 — Partitioning around mediods (PAM) analysis of 5-aza regulated genes in control NT2/D1-R1 and DNMT3B knockdown (sh-3B) cells. (TIF) [file pone.0053003.s004.tif]

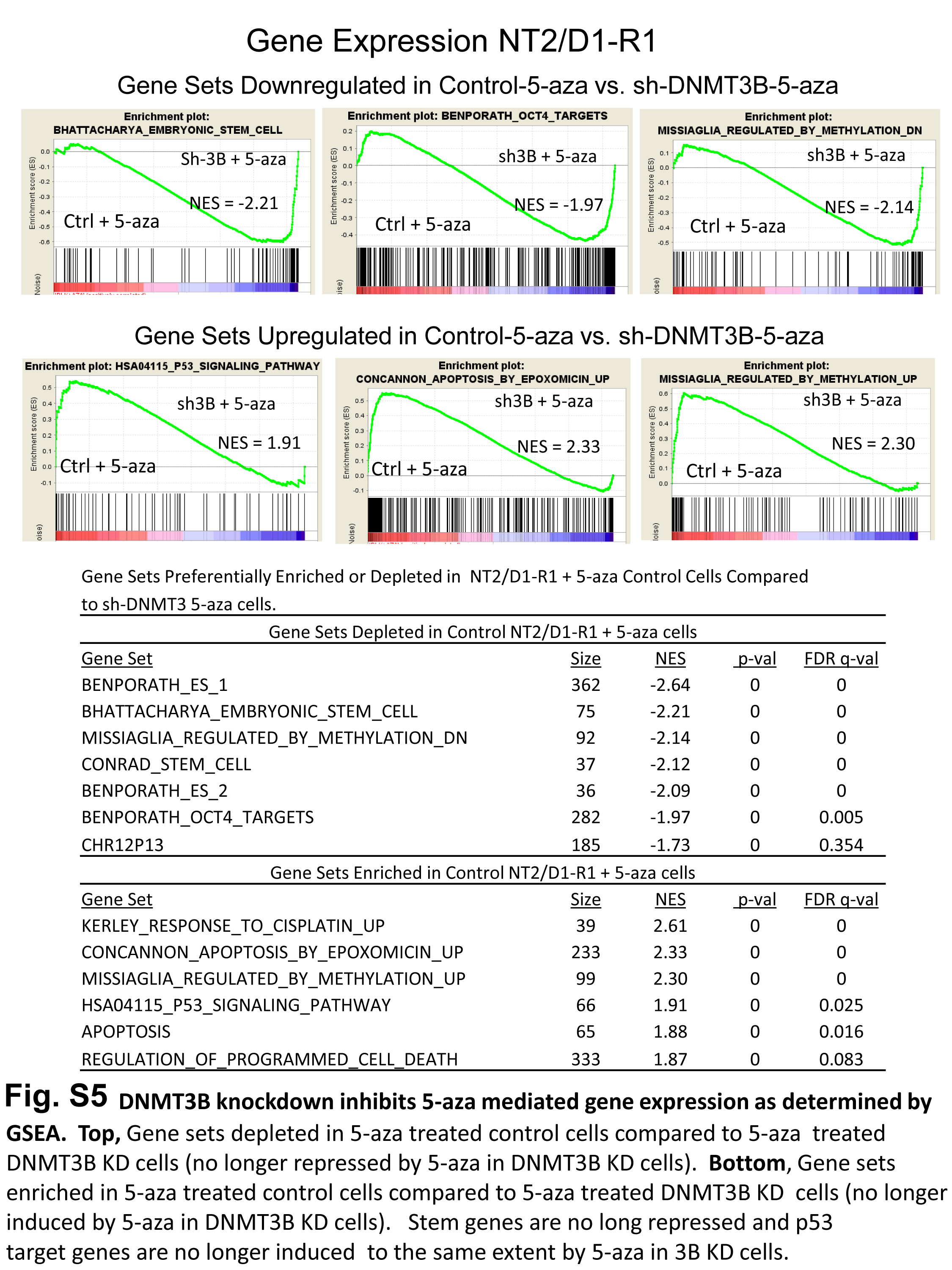

Supplement: Figure S5 — DNMT3B knockdown inhibits 5-aza mediated gene expression as determined by GSEA. Top, Gene sets depleted in 5-aza treated control NT2/D1-R1 cells compared to 5-aza treated DNMT3B KD cells (no longer repressed by 5-aza in DNMT3B KD cells). Bottom, Gene sets enriched in 5-aza treated control cells compared to 5-aza treated DNMT3B KD cells (no longer induced by 5-aza in DNMT3B KD cells). (TIF) [file pone.0053003.s005.tif]
